# Application of Combined Irradiation Mutagenesis Technique for Hyperproduction of Surfactin in *Bacillus velezensis* Strain AF_3B

**DOI:** 10.1155/ijm/5570585

**Published:** 2025-02-20

**Authors:** Syeda Amna Farooq, Shazia Khaliq, Saeed Ahmad, Neelma Ashraf, Muhammad Afzal Ghauri, Munir Ahmad Anwar, Kalsoom Akhtar

**Affiliations:** ^1^Industrial Biotechnology Division, National Institute for Biotechnology and Genetic Engineering, Constituent College, Pakistan Institute of Engineering and Applied Sciences, Faisalabad, Pakistan; ^2^Composting Research Group, Department of Chemical, Biological and Environmental Engineering, Escola d'Enginyeria, Universitat Autònoma de Barcelona, Barcelona, Spain; ^3^Institute of Pharmaceutical Sciences, Pharmaceutical Biology and Biotechnology, Albert-Ludwig University of Freiburg, Freiburg im Breisgau, Germany

## Abstract

The major challenge in large-scale industrial use of lipopeptide surfactin is the low yield by indigenous bacterial strains and the higher cost of its production that have been proved as a limiting factor in commercial applications. Therefore, there is an urgent demand for high-yielding strains that can be achieved through strain improvement. A first report on the use of a combination of UV and gamma-irradiation mutagenesis for the development of surfactin hyperproducing mutants of *Bacillus* spp. proved to be significant and resulted in a twofold enhancement in surfactin yield. The mutant was able to grow and produce surfactin on all the tested carbon and nitrogen sources, while 2% glycerol favored maximum surfactin yield (1.62 g/L) as compared to the wild-type strain that showed a maximum 0.85 g/L surfactin yield at 3% sucrose. Additionally, the mutant exhibited a good yield of pure surfactin, that is, 1.55 g/L as compared to the wild strain (0.411 g/L) by using corn steep liquor as the main component of the fermentation medium. The study concluded overall a threefold enhancement in the relative abundance of purified surfactin and its isoforms detected by matrix-assisted laser desorption/ionization time-of-flight (MALDI-TOF) analysis in mutant strain AF-UV*γ*2500.

## 1. Introduction

The demand for microbial biosurfactants (BSs) has been increasing gradually due to the ever-increasing environmental concern caused by the use of synthetic surfactants. These biological compounds enhanced biodegradability, lowered toxicity, and increased tolerance of extreme temperatures, pH, and saline environments [1]. BSs can be of different types, cyclic lipopeptides (LPs) being one of the significant moieties, produced by various *Bacillus* species [2]. Since the beginning, *Bacillus* species have been vastly studied for the production of LPs such as surfactin, iturin, and fengycin; their homologues; and isoforms that differ on the basis of fatty acid chain length and composition of amino acid in the peptide portion [3, 4]. They have a diverse number of applications in the fields of bioremediation as emulsifying and foaming agents, the biomedical industry as therapeutics, and the petroleum industry. They also have antiadhesive properties and have found various applications in pharmaceuticals, cosmetics, and the food industry.

LPs, despite being discovered 70 years earlier, are emerging bioactive molecules with several applications in a number of fields. LPs are reported to be produced by 11 different genera of bacteria and fungi, but *Bacillus* is the most studied among all due to naturally higher productivity in wild-type strains [5]. Commercial production of BSs has been reported in the literature, but the need of the hour is to develop a more sustainable and cheaper process for microbial BS production using low-cost raw materials, superficially agroindustrial wastes as substrate [6]. Owing to low production efficiency and high production cost, LP BSs cannot compete with synthetic chemical surfactants. This strategy can lower the overall capital of the process, accounting for only 10%–30% of the cost for raw materials used, along with solving major environmental and industrial concerns of pollution [4]. Genetic engineering of the producer strain and optimization of fermentation parameters such as temperature, pH, carbon-to-nitrogen ratio, and agitation rate are equally significant for increasing yield [7–10].

Surfactin holds an exceptional position in LPs due to its surface activity at very low concentrations as well as surface tension that can decrease from 72 to 27 mN m^−1^, hence called a BS. Surfactin is capable of having an antifungal activity against *Aspergillus* spp., *Fusarium* spp., *Botrytis cinerea*, *Sclerotinia sclerotiorum*, *Colletotrichum sclerotiorum*, and *Phoma complanata* [11]. Synthetic pesticides, which pose harmful environmental impacts and persistence of resistant pests, need to be replaced by these biological control compounds, which on the other hand are environmentally friendly [12]. Surfactin is also active against multidrug-resistant bacteria, for example, multidrug-resistant *Staphylococcus aureus* (MRSA) and *Pseudomonas aeruginosa* [13]. The antiviral and anticancer activities of surfactin have also been reported [14].

Many BSs, comprising mainly of those from *Bacillus* spp., are produced by nonribosomal peptide synthetases (NRPSs) and polyketide synthases (PKSs) that are multifactorial proteins with modular organization. Genus *Bacillus* contains a large diversity of these enzymes, and new strains could be exploited from the native environment to find BSs with novel properties [15].

Possible enhancement strategies for improving BS yield, that is, by selecting an economical substrate, optimizing the fermentation process, and using an efficient product recovery method, cannot alone be profitable and viable in terms of enhancement unless the capacity of the producer organism to synthesize the desired product with increased yield is genetically high. Hence, the capacity to produce a metabolite is greatly influenced by the organism's genetic assembly, which can further be improved by optimizing the fermentation process [16]. A conventional method of improving a producer strain is based on random mutagenesis, followed by screening to select hyperproducers among the viable survivors. This can be executed by chemical mutagens such as ethyl methane sulphonate, transposons, or radiations. Ionizing radiations, for example, gamma radiations, are categorized under physical mutagens, possess higher energy density than UV rays, and result in intense genetic alterations related to increased production of metabolites in microorganisms [17]. Genetically improving the strain has the benefit of long-term stability of the strain over improving other physiological parameters. Mulligan, Chow, and Gibbs reported the use of UV radiation for developing a stable *Bacillus subtilis* mutant, resulting in higher surfactin production than the wild-type strain [18]. The findings of Manikandan et al. [19] prove that gamma irradiation mutagenesis can improve the biocontrol characteristics of *Bacillus* spp. The rationale for opting for random mutagenesis over other enhancement strategies is the rapid process and efficient results with the stability of mutants. Zhang et al. reported a genome-reduced strain of *Bacillus amyloliquefaciens* LL3 by deletion of unnecessary genomic regions with the final surfactin yield of 311.35 mg/L [20]. However, a *B. subtilis* strain with a 10% genome deletion synthesizing plipastatin, bacilysin, toxins, and prophage reported a lower surfactin production [21]. In addition to this, NRPS genes are under the control of transcription regulatory genes. A study reported the inhibition of the comQXP locus leads to the downregulation of *srfA* genes, ultimately inhibiting surfactin production [22]. The use of artificial inducible promoters has been reported earlier in *B. subtilis* to increase surfactin production by 17-fold [23].

The purpose of the present study was to develop a mutant of indigenously isolated, surfactin-producing wild-type *Bacillus velezensis* strains using a combination of UV and gamma irradiation mutagenesis. Since this physical mutagen's combination has not been reported earlier for the enhancement of LPs or surfactin yield in *Bacillus*, followed by medium optimization to make the process economically feasible.

## 2. Experimental Procedures

### 2.1. Microorganisms and Medium Composition

Surfactin-producing *Bacillus* strains were isolated from soil rhizosphere around the roots of wheat and cotton plants growing in agricultural fields of the National Institute for Biotechnology and Genetics Engineering (NIBGE), Faisalabad, and oily sludge from the Attock petroleum oil refinery, Rawalpindi, using the method described by Farooq et al. [24]. Initially, the purified bacterial isolates were cultivated on Luria-Bertani medium at 37°C for 72 h. For the production of surfactin, a combination of minimal salt medium (MSM) and trace element solution was used with the following composition: 12 g/L sucrose, 5 g/L sodium glutamate, 1 g/L yeast extract, 1 g/L K_2_PO_4_, 0.5 g/L KCL, 0.5 g/L MgSO_4_.7H_2_O, 0.0016 g/L CuSO_4_, 0.0012 g/L Fe_2_(SO_4_)_3_, and 0.0004 g/L MnSO_4_, and pH was adjusted to 7.0 with 2 M HCl or NaOH. Media was autoclaved for 20 min at 15 Pa and 121°C.

### 2.2. Screening of Purified Isolates for the Production of LP

Purified bacterial isolates were screened for the production of LP (surfactin) by using the following techniques.

#### 2.2.1. Hemolytic Activity

For the analysis of hemolytic activity, 2 *μ*L of overnight broth of each isolate was overlaid on a blood agar plate and allowed to incubate for 72 h at 37°C. Colony growth on blood agar plates was observed with halo zone formation after 12–72 h and recorded as described previously [25].

#### 2.2.2. Oil Spreading Technique

The oil-spreading technique was implemented to assess the ability of isolates to produce surfactin as described previously [26]. This technique is based on the potential of BSs to vary the contact angle at the oil-aqueous interface. In this method, 15 mL of water was taken in a petri dish (diameter: 100 mm), to which 20 *μ*L of soybean oil was added, forming a thin layer of oil film over the surface of the water. Cell-free supernatant (10 *μ*L) of bacterial culture was gently poured into the center of the oil layer. After 30 s, the oil layer displacement to form a clear zone in the center was observed, which showed the presence of LPs. The diameter was measured, and the area was expressed in BS [18] units. One (BS) unit is the amount of surfactin, which can form a 1 cm^2^ area through displacement of oil [27].

#### 2.2.3. Emulsification Assay (*E*_24_)

The emulsification potential of each isolate was measured by the emulsification index “*E*_24_.” This assay was performed by the addition of soybean oil (2 mL) into cell-free supernatant (1 mL). Then, the mixture of oil and supernatant was subjected to a vortex for 5 min to mix them well. A negative and a positive control were performed by using an uninoculated culture medium and Tween 80, respectively. At hourly time intervals for 24 h, the emulsification activity was recorded by the following formula.

 $$\begin{array}{c} E_{24}=\left(\frac{\text{Total height of the emulsion layer}}{\text{Height of the aqueous layer}}\right)\times 100 \end{array}$$

#### 2.2.4. Drop Collapse Assay

To analyze the presence of BSs, a drop collapse assay was performed by the method described previously [28]. 96-well microplate wells were poured with crude oil, which was equilibrated for 24 h. Cell-free supernatant (5 *μ*L) was poured into the center of crude oil-filled wells, and after 1 min, the resulting drop size and shape were recorded with the help of a magnifying glass. Drop size having diameters larger than 1 mm and a flattened shape showed the presence of BS activity, while a round-shaped drop was considered a negative for BS activity. Triton X-100 (1 mg/mL) was used as a positive control and distilled water as a blank in the assay.

#### 2.2.5. Thin Layer Chromatography (TLC)

To confirm the production of surfactin in the fermentation medium by the selected isolates, TLC was performed using the silica gel plates as the stationary phase. The developing solvent system (mobile phase) used was methanol:chloroform:water (25:65:4) (V:V:V) and staining with the nonspecific reagent KMnO_4_ solution and ninhydrin [29].

### 2.3. Identification of Selected Isolates

Amplification of 16S rRNA, sequencing, and BlastN analysis were performed for the identification of selected surfactin-producing strains [30].

### 2.4. Mutant Development

#### 2.4.1. UV Irradiation Mutagenesis

Irradiation mutagenesis for mutant development was done using the protocol mentioned earlier [31] with some modifications. Selected *Bacillus* strains were grown in MSM at 180 rpm and 37°C for 15 h. The broths were diluted by normal saline (OD_600_ 0.1) and subjected to UV irradiation using a UV stratalinker (*λ* = 300) bulb at a fixed distance of 50 cm for 20, 40, 60, 80, 100, and 120 s. All the UV manipulations were carried out in the dark on a clean bench to avoid photoreactions. All experiments were performed in triplicates.

#### 2.4.2. Determination of Survival Ratios

After UV mutagenesis, the resultant broths were spread on Luria broth (LB) agar plates and allowed to incubate at 37°C for 24 h. After which colonies were counted, including the control plate, and the survival rate was calculated using the following equation:

 $$\begin{array}{c} \text{Survival rate}=\frac{\text{Surviving colonies on each plate}/\text{dose}}{\text{Total colonies on control plate}}\times 100 \end{array}$$

#### 2.4.3. Determination of Antifungal Activity

Potent colonies from UV irradiation were screened by an antifungal assay using the agar well diffusion method [32] for the qualitative analysis of the antimicrobial activities of LPs produced by wild-type and UV-irradiated mutant strains. Different fungal strains, including *Aspergillus niger*, *Aspergillus fumigatus*, *Aspergillus flavus*, and *Fusarium oxysporum*, were used as test strains for determining the antifungal potential of isolates. The fungal test strains were cultivated on potato dextrose (PD) agar medium as grams per liter: 1 g/L K_2_HPO_4_, 2 g/L NaNO_3_, 0.5 g/L MgSO_4_, 0.5 g/L KCl, 5 g/L yeast extract, and 30 g/L dextrose with pH adjusted to 6.5. Spore suspension (0.1 mL) of the test strains was aseptically spread onto PD agar plates. The cavities (3–4 mm) were molded using the rear end of the sterile 1000 *μ*L micropipette tip and were filled with the cell-free supernatant (100 *μ*L) of wild-type and UV-irradiated bacterial cultures [33]. The results were recorded after an incubation time of 2–3 days at 30°C ± 2°C. All antifungal assays were carried out in triplicates.

#### 2.4.4. *γ*-Irradiation Mutagenesis

Selected UV-irradiated survivors were taken in 10 mL glass bottles (5 mL spore suspension), and wild-type strains were irradiated with gamma rays from a source of cesium (Cs^139^, Nuclear Institute of Agricultural Biotechnology (NIAB), Faisalabad) using six multiple doses: 500, 1000, 1500, 2000, 2500, and 3000 Gy at room temperature. Survival ratios were again calculated as described above.

### 2.5. Screening of Selected Survivors With Enhanced Surfactin Yield

The survivors that appeared on agar plates after irradiation mutagenesis were selected randomly along with phenotypically changed colonies, if any, and screened by using the abovementioned techniques to find out mutant colonies with enhanced ability to synthesize LP surfactin as compared to wild-type strains. The mutant colonies with hyperproduction of crude LPs were selected for optimization studies along with their wild-type strain.

### 2.6. Optimization of Different Fermentation Parameters

#### 2.6.1. Effect of Carbon and Nitrogen Sources

To identify the best carbon source for the production of surfactin by the selected hyperproducer mutant and wild-type strains, different carbon sources (sucrose, glucose, maltose, fructose, lactose, glycerol, and soluble starch) at the concentration of 1% *w*/*v* were used in the production medium while the other constituents remained the same. Then varying the concentrations (1%, 2%, 3%, 4%, and 5%) of the best carbon source was also used to determine the optimum concentration of the selected carbon source. Cultures were harvested after 72 h and analyzed for antifungal activity, surfactin yield, dry cell mass, and other parameters. For the identification of the best nitrogen source, different nitrogen sources (yeast extract, peptone, ammonium sulphate, ammonium nitrate, ammonium chloride, sodium nitrate, and urea) at 0.3% (*w*/*v*) concentration were added to the medium containing the optimized carbon source. Fermentation experiments were performed at 37°C and 180 rpm for 3 days, harvested, and analyzed.

#### 2.6.2. Effect of pH and Temperature

To investigate the effect of initial pH on surfactin yield, different pH ranges (4.0, 5.0, 6.0, 7.0, 8.0, and 9.0) of fermentation medium were used to find out the optimum pH. Likewise, a temperature range from 25°C to 45°C (25°C, 30°C, 35°C, 37°C, 40°C, and 45°C) was maintained for the incubation of fermentation medium to select the optimum temperature in order to achieve hyperproduction of surfactin.

#### 2.6.3. Effect of Inoculum Size (*V*/*V*)

Different inoculum sizes (2.5%, 5%, 7.5%, and 10% *v*/*v*) were tested to study the effect of inoculum on surfactin yield, while the optimized values of other fermentation parameters were used.

#### 2.6.4. Effect of Incubation Period

To investigate the effect of the incubation period on the productivity of surfactin, the inoculated medium was incubated for 5 days, and samples were taken after regular time intervals and subjected to analysis.

### 2.7. Evaluation of Different Agroindustrial Wastes as Substrate

Aqueous extracts of different agroindustrial wastes (molasses, corn steep liquor, orange peel, banana peel, and sugarcane bagasse) at the concentration of 7.5% (*v*/*v*) were tested to find an alternative of refined carbon and nitrogen sources in the fermentation medium by keeping all other salts at their basal levels. To prepare the stock solution of agricultural waste 10% (*w*/*v*), suspensions for each dry residue were filtered through a muslin cloth and Whatman filter paper No. 1, and filtrates of the extracts were autoclaved.

### 2.8. Extraction and Purification of LP-Surfactin

Cultures were harvested and centrifuged (Beckman Coulter centrifuge) for 20 min at 10,000 rpm and 4°C. The supernatants were acidified by adjusting pH to 2.0 and were kept at 4°C overnight for the precipitation of LPs. Crude LPs concentrated on the upper layer were collected through centrifugation and freeze-dried. The concentrated LP collected from wild-type and mutant strains was weighed, resuspended in Milli-Q water, and sterilized using a 0.45 *μ*m Durapore syringe filter membrane.

### 2.9. Purification of LPs by Column Chromatography

The crude extracts in Milli-Q water were desalted through C_18_ reversed-phase column chromatography. Samples were applied to the column and washed with Solvent A (Milli-Q-0.1% TFA (trifluoroacetic acid)) to get rid of salts, if any. The mobile phase consisted of 95% acetonitrile-0.1% TFA (Solvent B), which was applied to collect the entire extract as a single elution and then finally subjected to high-performance liquid chromatography (HPLC). A two-step gradient was set with Solvent B from 5% to 40% for 10 min and from 40% to 95% for 30 min at a flow rate of 1 mL/min for compound purification. A wavelength of 214 nm was set using a UV detector to monitor the eluted compounds. All the peaks were collected individually in order to separate out various LPs in the extract for analysis at matrix-assisted laser desorption/ionization time-of-flight (MALDI-TOF).

### 2.10. Identification of Surfactin by Mass Spectrometric Analysis

#### 2.10.1. Matrix-Assisted Laser Desorption/Ionization Time of Flight

For the analysis of mass spectra, column-purified, HPLC-eluted LPs with acetonitrile-0.1% TFA (1 *μ*L) were individually applied to the target spot on the MALDI plate, dried, and covered with (0.5 *μ*L) matrix medium, specifically, 5 mg/mL of *α*-cyano-4-hydroxycinnamic acid dissolved in 60% acetonitrile (0.1% TFA). The samples were air-dried again and analyzed by a Voyager DE PRO MALDI-TOF mass spectrometer (Applied Biosystems), and monoisotopic masses were recorded using positive ion and reflector mode, as described previously [34–36], with some modifications.

## 3. Results

### 3.1. Isolation and Identification

**Figure 1:**
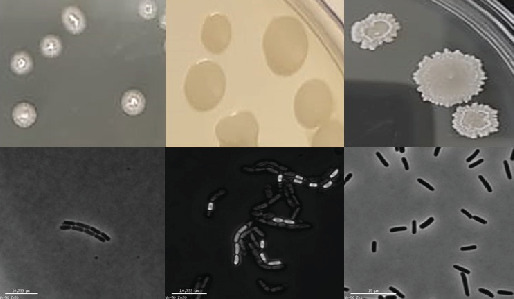
Colony morphology and microscopic examination of selected indigenous isolates. Left to right: isolate AF_3B, isolate BS9, and isolate OS2.

**Figure 2:**
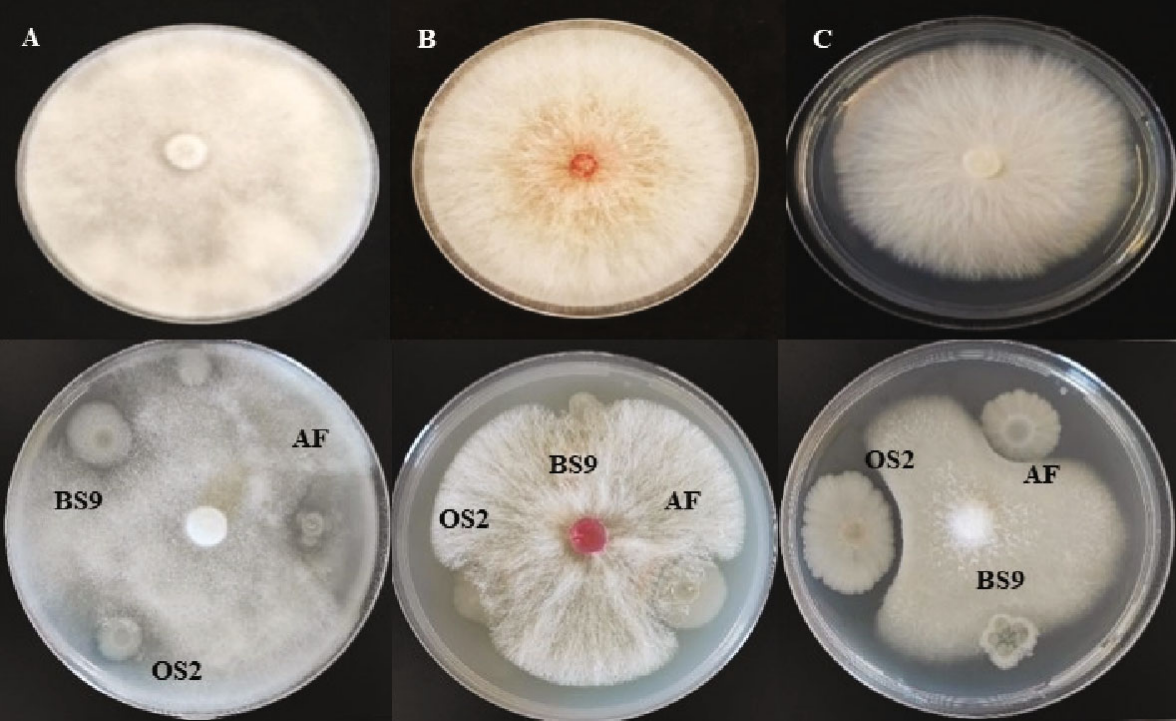
Antifungal activity of *Bacillus* isolates. (A) *Pythium ultimum*, (B) *Fusarium culmorum*, and (C) *Fusarium oxysporum*.

The bacterial colonies that appeared on solid agar plates were picked randomly based on their *Bacillus*-like colony morphology. The shape and size indicated circular to elongated colonies with wrinkled margins, and the color observed was white, off-white, creamy, and pale with variation in pigmentation. Elevation in colonies was observed as flat, convex, and undulating. Among 50 wild bacterial colonies that were isolated and purified from the soil and oil samples, only three bacterial isolates (AF_3B, BS9, and OS2) were selected for further studies (Figure 1) on the basis of dominance in antifungal activity (Figure 2). The coding and numbering of the isolates were based on the location, source, order, and nature of the sample collected. Taxonomical identification of selected isolates was performed by amplification, sequencing, and BlastN analysis of 16S rRNA. The isolate AF_3B (OL771696.1) and OS2 (OL771697.1) were identified as *B. velezensis* by showing more than 99% identity with *B. velezensis* strain FZB42, while BS9 (OL757572.1) was identified as *B. amyloliquefaciens* due to its 99.73% identity with *B. amyloliquefaciens* strain NBRC 15535.

### 3.2. Screening for LP Production

**Figure 3:**
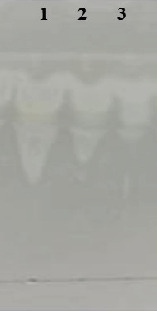
Thin layer chromatographic analysis of cell-free supernatant of selected isolates. (1) *Bacillus* strain AF_3B, (2) *Bacillus* strain BS9, and (3) *Bacillus* strain OS2.

Selected isolates were further tested for hemolysis of red blood cells, oil spreading, drop collapse, and emulsification potential. All three isolates exhibited good activities regarding these assays, but isolate AF_3B was found more efficient as compared to OS2 and BS9 (Table 1). Moreover, TLC results revealed that these strains are predominantly producing LP surfactin, as shown in Figure 3.

### 3.3. Irradiation Mutagenesis

All three wild isolates were subjected to irradiation mutagenesis in order to procure surfactin hyperproducing mutant strains.

**Figure 4:**
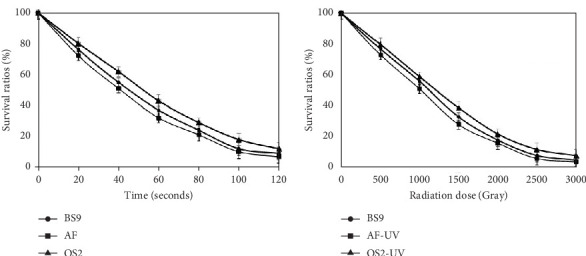
Survival rates after irradiation mutagenesis. (a) Survival of bacterial cells after first UV radiation exposure and (b) survival of bacterial cells after second gamma radiation exposure.

Spore suspensions of selected *Bacillus* isolates were exposed to various doses of UV radiation, and the percentage survival rates of each isolate were calculated at each exposure. The survival rates of 10% and 7% of the viable cells were observed at 100 and 120 s UV exposure, respectively (Figure 4(a)), and maximum colonies were picked randomly at these exposures. In addition to this, after 100 s of UV exposure, 90% of the cells had died, and the percentage exceeded 93% after 120 s. About 10 colonies of each isolate at the maximum time span of UV radiation that resulted in 90% and 93% cell death were randomly selected and evaluated for preliminary analysis through antifungal activities. Only two mutant colonies, one from each UV-treated isolate AF_3B coded as AF-UV and OS2 (OS2-UV) isolates were found to exhibit enhanced antifungal activities as compared to wild-type strains of respective isolates. On the other hand, no colony of BS9 appeared on agar plates after spreading UV-treated spore suspensions. The two mutant strains as well as the wild-type strain of BS9 were subjected to a second round of irradiation mutagenesis by using a gamma rays' source of cesium (Cs^139^). The survival ratio of each mutant colony and wild-type strain BS9 at each successive dose of gamma radiation was also calculated (Figure 4(b)). Gamma irradiation of 1500, 2000, 2500, and 3000 Gy resulted in 10%, 7%, 6%, and 4% survival ratios for all the treated strains.

### 3.4. Effects of Irradiation Mutagenesis on Colony Morphologies

**Figure 5:**
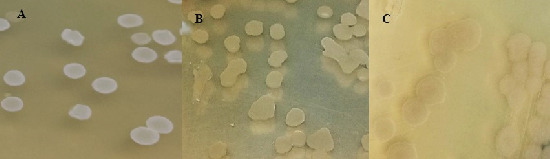
Colony morphology after irradiation mutagenesis. (A) Colony morphology of wild-type strain AF_3B, (B) colony morphology of mutant strain AF-UV*γ*1500, and (C) colony morphology of mutant strain AF-UV*γ*2500.

The morphology of the isolated mutants was opaque and circular, and no unusual phenotypes were noticed in the case of UV-treated *Bacillus* isolates. But gamma-irradiated spore suspension of mutant AF-UV at 1500 and 2500 Gy that resulted in the 10% and 6% survival rates displayed the appearance of two morphological variants named AF-UV*γ*1500 and AF-UV*γ*2500 with slight changes in color and colony morphology, an increase in size (3–4 mm in diameter), and raised/undulated margins on LB agar plates in Figures 5(B) and 5(C). Including these two morphological mutants, a total of 180 bacterial colonies—60 for each UV-treated mutant (AF-UV and OS2-UV) and wild-type isolate BS9—were randomly picked at those doses of gamma radiation that caused 10% or less than 10% survival of cells and screened for the production of BSs by following assays.

### 3.5. Screening for the Production of LPs

#### 3.5.1. Hemolytic Activity

Out of 180 colonies tested, 50 colonies were found positive for hemolytic activity. Among positive colonies, 30 belonged to UV mutant (AF-UV), 15 belonged to UV mutant (OS2-UV), and five belonged to wild-type isolate BS9 (Table S1). Mutant colonies designated as AF-UV*γ*2500 and AF-UV*γ*1500 showed the largest halo zone (1.8 and 1.67 cm) formation as compared to others (Table 1).

#### 3.5.2. Drop Collapse Assay

All 50 colonies positive for hemolysis were further subjected to a drop collapse assay, and it was found that mutant strains AF-UV*γ*1500, AF-UV*γ*2500, OS2-UV*γ*2500, and OS2-UV*γ*3000 were significantly showing higher values for the drop collapse assay (Table 1).

#### 3.5.3. Oil Displacement Activity

The results obtained by the oil displacement assay were found to be similar to those of the results of the drop collapse assay. The maximum zone formation was exhibited by mutant AF-UV-*γ*2500 (Table 1).

#### 3.5.4. Emulsification Activity

The emulsification activities were observed by all five mutants, but mutant AF-UV*γ*2500 and AF-UV*γ*1500 exhibited a greater emulsification index (*E*_24_) as compared to all other mutants as well as wild-type strains. The emulsification index has been given in Table 1, according to which it has been concluded that AF-UV*γ*2500 and AF-UV*γ*1500 were more potent regarding the production of LP BS, particularly surfactin (Table 1). An uninoculated culture was used as a negative control, and Tween 80 was used as a positive control.

#### 3.5.5. Antifungal Activity

Subsequently, the antifungal activity of AF-UV*γ*2500, AF-UV*γ*1500, OS2-UV*γ*2500, and OS2-UV*γ*3000 was also evaluated against *A. flavus* and *F. oxysporum*. The diameter of the zone of inhibition produced by AF-UV*γ*2500 showed significant differences (*p* < 0.01) compared with other mutants and the wild-type strains (data not shown). On the basis of these observations, only mutant AF-UV*γ*2500 was selected for optimization studies.

### 3.6. Optimization Studies

#### 3.6.1. Effect of Carbon and Nitrogen Sources

**Figure 6:**
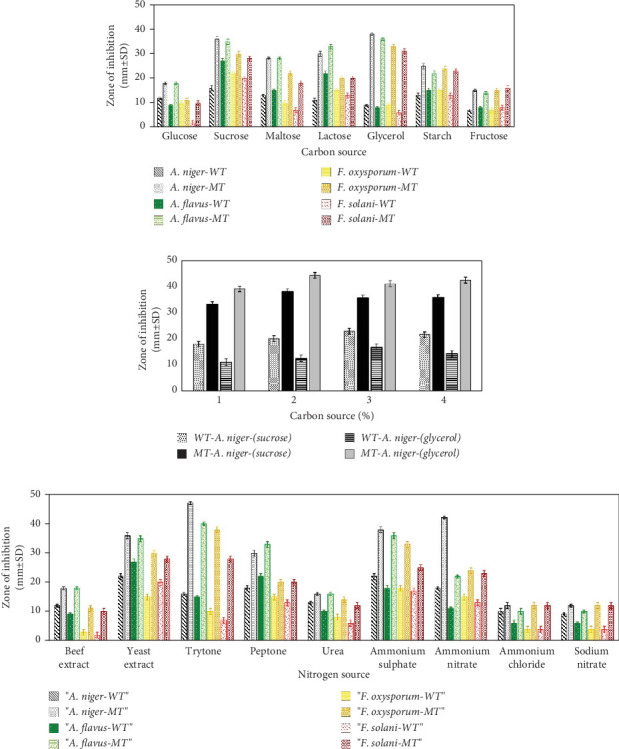
Optimization of carbon and nitrogen source. (a) Effect of different carbon sources at 1% (*w*/*v*) concentration on maximum antifungal activity. For wild-type strain AF_3B (WT) and mutant strain AF-UV*γ*2500 (MT) against *A. niger*, *A. flavus*, *F. solani*, and *F. oxysporum* after 72 h of incubation. (b) Effect of sucrose and glycerol carbon concentration (*w*/*v*) on maximum antifungal activity. For wild-type strain AF_3B (WT) and mutant strain AF-UV*γ*2500 (MT) against *A. niger*, *A. flavus*, *F. solani*, and *F. oxysporum* after 72 h of incubation. (c) Effect of different nitrogen sources at 0.3% (*w*/*v*) concentration on maximum antifungal activity. For wild-type strain AF_3B (WT) and mutant strain AF-UV*γ*2500 (MT) against *A. niger*, *A. flavus*, *F. solani*, and *F. oxysporum* after 72 h of incubation.

In the current study, the effect of different carbon and nitrogen sources on the growth and production of surfactin by the wild-type isolate (AF_3B) and its mutant (AF-UV*γ*2500) was evaluated. Both the strains were allowed to grow and produce LP surfactin in the presence of a 1% concentration of each carbon source. The cultures were harvested after 72 h of incubation at 37°C and checked for antifungal activity. The maximum antifungal activity by the wild-type strain (16 ± 1.2 mm diameter of the zone of inhibition) was observed in the presence of 1% sucrose as compared to the mutant AF-UV*γ*2500, which showed maximum antifungal activity (39 ± 2 mm diameter of the zone of inhibition) at a 1% glycerol concentration in the fermentation medium (Figure 6(a)). The mutant AF-UV*γ*2500 also showed higher antifungal activities in the presence of glycerol (37 ± 1.8 mm), lactose (30 ± 1.6 mm), maltose (28 ± 1.1 mm), starch (25 ± 1.3 mm), and glucose (18 ± 1.2 mm) as compared to the wild-type strain. However, very low values of antifungal activity were obtained by the wild-type strain when glycerol was used as a carbon source.

The maximum dry cell mass (3.81 and 4.98 g/L) was obtained for both wild-type (AF_3B) and mutant (AF-UV*γ*2500) strains at 1% sucrose and glycerol, respectively, and the highest amount of surfactin (1.02 g/L) for mutant AF-UV*γ*2500 was also obtained in the presence of glycerol, followed by sucrose (0.99 g/L). However, the wild-type strain AF_3B exhibited the highest values of surfactin (0.65 g/L) with sucrose as a carbon source. The other parameters related to the production of surfactin, such as the emulsification index by mutant AF-UV*γ*2500, ranged from 49% to 76%, depending on the different carbon sources used. The highest value of emulsification index (76%) by mutant AF-UV*γ*2500 was also observed when glycerol was used as the sole carbon source, followed by sucrose (74%). Alternatively, the wild-type strain AF_3B exhibited the highest values of emulsification index (46%) in the presence of sucrose (Table 2).

Mutant (AF-UV*γ*2500) and wild-type strain (AF_3B) were also cultivated on different concentrations (1%–5%) of optimized carbon sources, for example, glycerol and sucrose, respectively. It was found that a 2% concentration of glycerol favored the maximum antifungal activity (44 ± 1.2 mm) against *A. niger* in the case of the mutant (AF-UV*γ*2500), while the wild-type strain (AF) exhibited the highest antifungal activity (19 ± 10 mm) in the presence of 3% sucrose (Figure 6(b)).

The mutant AF-UV*γ*2500 and wild-type strain AF_3B were found to produce LP surfactin on all the tested nitrogen sources. There was an increase in antifungal activities against *A. niger* and *F. oxysporum* by the wild-type strain, and the diameter of the zone of inhibition reached 22 ± 1.2 mm (Figure 6(c)) with maximum cell mass (3.88 ± 0.030 g/L) and surfactin yield (0.75 ± 0.021 g/L) in the presence of 0.3% yeast extract (Table 3). While the mutant isolate (AF-UV-*γ*2500) showed maximum antifungal activities by producing a zone of inhibition of 47 ± 2 and 40 ± 1.5 mm against *A. niger* and *F. oxysporum*, respectively, along with maximum dry cell mass (5.25 ± 0.030 g/L) and surfactin yield (1.55 ± 0.061 g/L) when 0.3% tryptone was used as the nitrogen source. The highest emulsifying index (82.28% and 51.3%) was also observed when tryptone and yeast extract were used as nitrogen sources for mutant and wild-type strains, respectively.

#### 3.6.2. Effect of pH, Temperature, Inoculum Size, and Incubation Period

**Figure 7:**
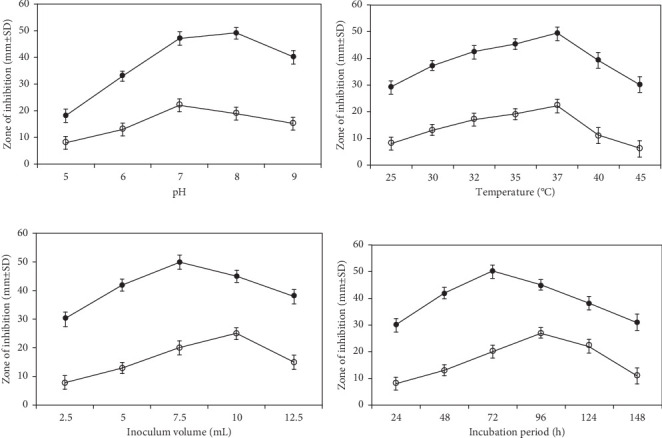
Optimization studies. (a) Effect of pH, (b) effect of temperature, (c) effect of inoculum size *v*/*v*, and (d) effect of incubation period on maximum antifungal activity of wild type (WT) strain AF_3B and mutant (MT) strain AF-UV*γ*2500 against *Aspergillus niger*.

The effect of the initial pH of the fermentation medium on the production of surfactin by mutant and wild-type strains was also studied while the medium was supplemented with optimized carbon and nitrogen sources. There was a slight increase in antifungal activity against *A. niger* by mutant isolate AF-UV*γ*2500 at pH 8.0, and the diameter of the zone of inhibition reached 49 ± 2.10 mm. The wild-type strain AF_3B exhibited the maximum zone of inhibition against *A. niger* at pH 7.0 (Figure 7(a)). For wild-type and mutant strains, a temperature of 37°C was found optimum with a maximum zone of inhibition (Figure 7(b)). In all previous experiments, 5% inoculum (*v*/*v*) was used. After applying a range of inoculum volumes (2.5%–12.5%), the highest antifungal activity (50 ± 2.31 mm) against *A. niger* was observed by mutant isolate AF-UV*γ*2500 at 7.5% inoculum volume, while the wild-type strain showed maximum activity (25 ± 1.72 mm) when 10% inoculum was used (Figure 7(c)). The cultures were harvested after regular intervals of time from 24 to 144 h, and antifungal activities were determined. It was observed that an incubation period of 72 h resulted in the maximum zone of inhibition by mutant AF-UV*γ*2500 (50 ± 2.31 mm) (Figure 7(d)), while the wild-type strain exhibited a maximum zone of inhibition (27 ± 1.65 mm) after 96 h. The surfactin yield in terms of dry weight was determined after the optimization of all the parameters and a slight increase that led the production to 1.62 g/L in the mutant strain as compared to the wild type (0.85 g/L) (data not shown). Therefore, we can say that there is an early induction and increased yield of surfactin synthesis in the mutant strain as compared to the wild-type strain.

### 3.7. Evaluation of Different Agroindustrial Wastes as Substrate

**Figure 8:**
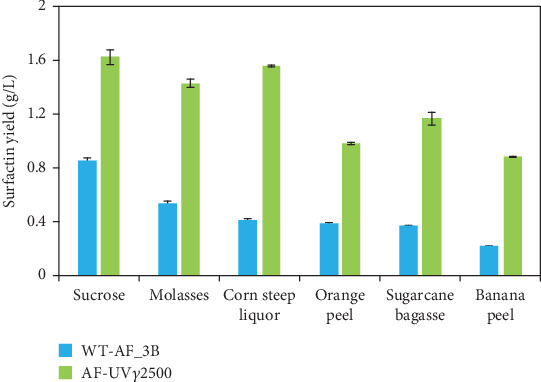
Effect of agroindustrial wastes as a substrate on surfactin yield (grams per liter).

At optimized conditions, different agroindustrial wastes were evaluated as substrates instead of using refined carbon and nitrogen sources. It was observed that wild-type strain AF_3B produced a maximum amount of surfactin (0.530 ± 0.017 g/L) when cultivated with molasses followed by corn steep liquor (0.411 ± 0.010 g/L) as compared to sucrose (1.62 ± 0.026 g/L), as shown in Figure 8. In contrast, the mutant AF-UV*γ*2500 produced a maximum of 1.55 ± 0.005 g/L surfactin when corn steep liquor was used as a substrate, and all other raw material sources were also found significantly useful for the production of surfactin.

### 3.8. Identification of LPs

**Figure 9:**
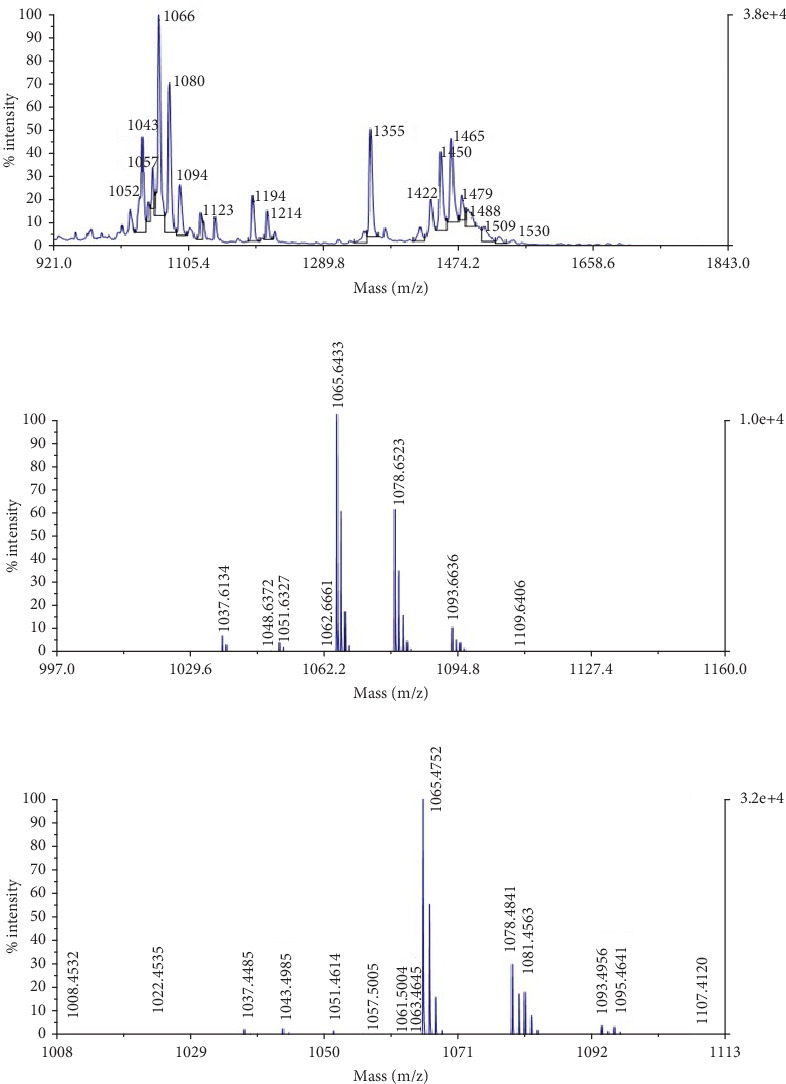
MALDI-TOF analysis of extracted lipopeptide. (a) Extracted metabolites from wild-type strain AF_3B without separation of respective lipopeptides, (b) in wild-type strain AF_3B after extraction and purification of surfactin lipopeptide, and (c) in mutant strain AF-UV*γ*2500 after extraction and purification of surfactin lipopeptide.

**Figure 10:**
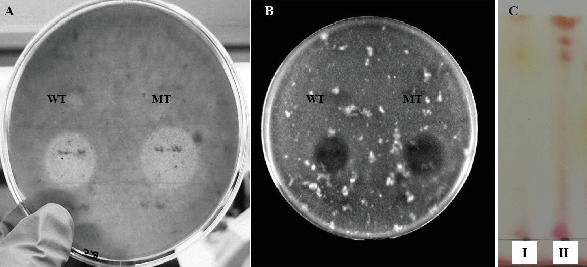
Antifungal activity assay of purified surfactin extract of wild type AF_3B (WT) and mutant AF-UV*γ*2500 (MT). (A) Against *Botrytis cinerea*, (B) against *Fusarium culmorum*, and (C) thin layer chromatography (TLC) of purified surfactin extract from (I) wild-type strain AF_3B and (II) mutant strain AF-UV*γ*2500.

In order to confirm the nature and concentration of LPs (surfactin) produced by wild-type and mutant strains, the purified extracts were subjected to investigation by MALDI-TOF analysis. Two families of metabolites were detected in the mass peaks range 1000–1550 m/z belonging to surfactins and plipastatins, and multiple isoforms of surfactin were identified as protonated precursor ions of sodium and potassium adducts, which actually are 1008, 1022, and 1078 m/z with a difference of 14 Da (Figure 9(a)). Moreover, the 1022 m/z precursor ion appeared as 1061 m/z as a result of the formation of a potassium adduct. The purified peaks of surfactin obtained by mutant and wild-type strains (Figures 9(b) and 9(c)) that were confirmed through MALDI-TOF analysis were also subjected to antifungal assay and TLC analysis. Halo zones formed by mutant AF-UV*γ*2500 were larger in diameter as compared to wild-type strain AF_3B (Figures 10(A) and 10(B)). Moreover, dense bands of surfactin and its isoforms were seen on the TLC plate indicating a greater concentration of surfactin, and visual separation of its isoforms was detected in mutant AF-UV*γ*2500 extract as compared to wild-type strain AF_3B (Figure 10(C)).

## 4. Discussion

The genome of *B. velezensis* devotes about 8%–10% of its whole genome to some significant biosynthetic gene clusters that are responsible for its antimicrobial potential related to the production of surfactin, fengycin, bacillomycin, bacillaene, plipastatins, difficidin, and bacillibactin. These compounds are synthesized under the control of the NRPS enzyme complex [37, 38]. BSs of LP nature have been continuously gaining attention for their properties and advantages. From the ease in synthesis by readily available microorganisms by using renewable carbon sources, appearing in a range of isoforms and chemical natures with varying applications to being active at low volumes and safe biodegradation in the environment. Hence, microbial BSs are considered the emerging green technology for the replacement of synthetic surfactants for their applications in cleaning, pharmaceuticals, food, and environmental restoration [39–41]. However, the production of surfactin on a large scale sets a problem of low yield and high cost. Their production cost is not affordable compared to their synthetic counterparts. As a result, the market demand is easily met by the availability of synthetic surfactants for daily use [42]. However, the use of synthetic surfactants poses harmful effects due to the presence of antifoaming characteristics and the toxic and corrosive nature of the compounds used in their composition [43].

However, the key limitation is low yield and high cost, as the average natural production of surfactin from wild-type strains has been reported to be 100–600 mg/L [44–46]. Therefore, the abovementioned facts urged us to conduct this study with the aim of the hyperproduction of surfactin from indigenous *Bacillus* strains by random mutagenesis using a combination of UV and gamma irradiation that has not been reported earlier. A *P. aeruginosa* MR01 mutant strain developed by gamma radiation mutagenesis resulted in a one and a half-fold rise in BS production [47]. During this study, it was observed that higher doses of UV and gamma radiations caused more than 90% cell death (Figure 4), and a few variants obtained at survival rates of lower than 10% exhibited more surfactin production as compared to the wild-type strains (Table 1). Moreover, the final round of mutagenesis of UV mutants along with gamma radiation resulted in the appearance of morphological mutants also when compared to the wild-type strain (Figure 5). This variation in the physical appearance of the colonies was in accordance with [48], where they reported that after the treatment of *Bacillus thuringiensis* var. *israelensis* with N-methy-N⁣′-nitro-N- nitroso guanidine (MNNG), the resultant mutants differed phenotypically from their wild-type strain. In addition to the change in colony morphology, mutations produced by gamma radiations were found to be irreversible and stable. Among randomly selected mutant colonies obtained at higher doses, both morphological mutants exhibited significantly higher potential for hemolytic, drop collapse, antifungal, and other related activities as compared to their wild-type strains (Table 1). These observations indicated that a combination of UV and gamma radiations had a greater impact as compared to the use of individual radiations on surfactin yield, and the hyperproducing mutant was also found stable in terms of morphology and activity throughout the study (data not shown). Previously, two potent mutants were developed from *B. subtilis* UTB1 through gamma irradiation with enhanced antifungal activity against *A. flavus* [49]. The oil displacement assay is considered an indicator assay for BS production because as the surfactant production increases, the oil–water interfacial tension decreases [50]. In our study, we report the highest oil displacement potential of mutant AF-UV*γ*2500 with a 3.1 cm oil displaced zone, which was 2.5 cm in its wild-type strain (Table 1). Similarly, emulsification properties and surface activities are the index for the production of surfactants in media [31]. Our data showed that the indigenous *Bacillus* isolates used in this study had naturally good emulsification potential that was further enhanced to 76% in the case of mutant AF-UV*γ*2500. This property depicts the enhancement in secondary metabolite surfactin production. The improvement in BS production from one mutant to another was the result of the regulation of bioproduct synthesis in the metabolic pathway [51]. Moreover, the antifungal potential of the wild-type strains was improved considerably after irradiation mutagenesis. We can relate the antifungal activity of the mutants with the results of previously reported studies [52, 53]. They reported a random ion beam-implanted mutant strain of *B. subtilis* with high antifungal activity against *Rhizoctonia solani* and *Gibberella zeae* as compared to the wild-type strain. One of the morphological as well as surfactin hyperproducing mutant AF-UV*γ*2500 was selected for optimization studies, and it was observed that sucrose and glycerol favored the maximum yield of surfactin on the basis of the zone of inhibition for the wild-type strain AF_3B (0.65 g/L) and mutant AF-UV*γ*2500 (1.02 g/L) (Table 2). The optimization of the concentration of selected carbon sources revealed that 3% sucrose and 2% glycerol favored the formation of a zone of inhibition with the largest diameter for wild-type and mutant AF-UV*γ*2500 strains, respectively (Figure 6(b)). Earlier, it has been reported that BSs were only produced in a medium containing dextrose as a carbon source, while other carbon sources like sucrose, galactose, xylose, and fructose were suitable for growth but not the production of BSs [54]. Whereas the mutant AF-UV*γ*2500 indicated the ability to produce surfactin and support maximum growth not only with glycerol but also on other carbon sources such as glucose (dry cell mass 3.81 g/L and surfactin yield 0.77 g/L). Similarly, the production of surfactin with 0.77 g/L yield has been reported with brown sugar and urea as carbon and nitrogen sources, respectively, from *Bacillus atrophaeus* 5-2a [55]. It was very interesting to observe that glucose supported good growth of bacteria, but the levels of surfactin yield and antifungal activity were low. This phenomenon indicated that the synthesis of surfactin by mutant AF-UV*γ*2500 is not totally growth-dependent, and there is also a shift in this mutant toward the use of raw materials as carbon sources, such as glycerol, as compared to the wild-type strain AF_3B. The relationship between the bacterial growth rate and concentration of the substrate utilized is of great significance as it dictates not only the optimum substrate for the system but also the inhibitory effect of the substrate [56].

On the other hand, among different nitrogen sources used, the maximum growth in form of dry cell mass (5.25 g/L) and surfactin yield (1.55 g/L) were favored by tryptone followed by (NH_4_)_2_SO_4_ (1.08 g/L) in case of mutant AF-UV*γ*2500 as compared to the wild-type strain AF_3B that produced maximum cell mass (3.88 g/L) and surfactin (0.75 g/L) in the presence of yeast extract. Formerly, some *B. subtilis* strains have been reported that could not use (NH_4_)_2_SO_4_ for growth or metabolite production; however, they were able to use KNO_3_, NaNO_3_, or NH_4_NO_3_ [57] as nitrogen sources. In this study, it was noted that mutant AF-UV*γ*2500 and wild-type strains were capable of growing and producing surfactin using all the tested nitrogen sources. These observations demonstrated that strains used in this study are more competitive than previously reported *Bacillus* strains for the production of surfactin and can be used for industrial-scale applications. The considerably higher emulsifying activities (82.28 and 51.3%) were observed when tryptone and yeast extract were used as nitrogen sources for mutant and wild-type strains, respectively, in comparison to already reported emulsifying activities of 43.3% and 41.0% for *Bacillus* strains using yeast extract and sodium nitrate as nitrogen, respectively [2].

On the basis of the zone of inhibition, it was concluded the initial pH of 8.0 and 7.0 supported maximum product formation for mutant and wild-type strains, respectively, whereas the maximum cell mass formation, surfactin yield, and zone of inhibition by both wild-type and mutant strains were obtained at 37°C. Similarly, the minimum inoculum required for the mutant was observed to be 7.5% with 72 h of incubation, whereas the wild-type strain required a minimum of 10% inoculum and a 96-h incubation period for best activity (Figure 7).

Using cheap raw materials as a source of nutrients for the growth of microorganisms and large-scale production of fermentation products is a common way to make an industrial experiment economically favorable. Hence, the selection of an ideal substrate is a key factor for the production of biomolecules, including BSs [58]. The mutant AF-UV*γ*2500 produced a maximum of 1.55 g/L surfactin with corn steep liquor as a substrate, and all other sources were also found fairly positive for the production of surfactin (Figure 8), which indicated the raw source utilization ability was improved in the mutant strain, and the production of a good yield of surfactin was also indicated by this mutant on all waste materials as compared to the wild-type strain. This also indicates that the utilization of agroindustrial waste as carbon (corn steep liquor) produced a comparable surfactin yield to a refined carbon source (sucrose 1.62 g/L). It has also been reported in an earlier study that a yield of surfactin 1.3 g/L was achieved using 10% (*v*/*v*) corn steep liqueur [59] as a substrate.

The MALDI-TOF spectra for the comparison between wild and mutant metabolites showed that the developed mutant AF-UV*γ*2500 was capable of producing multiple isoforms of surfactin as compared to its wild-type strain after mutagenesis, as illustrated in Figure 9(c). The mass spectra were well resolved, ranging within a mass-to-charge ratio of 1008–1095. The masses designated as 1008, 1022, 1030, 1036, 1043, 1051, 1065, and 1078 were found associated with the heptapeptide moiety and appeared as precursor ions of sodium and potassium adducts [60, 61]. Pecci et al. [3] reported a similar purification and characterization study of two sets of molecular ion species belonging to the surfactin and fengycin families of LPs and their isoforms from the *Bacillus licheniformis* V9T14 strain, while the wild-type strain was able to produce surfactin isoforms in the mass range of 1037–1093 m/z, but in very low relative abundance (Figure 9(b)). The relative abundance of the surfactin isoforms in mutant AF-UV*γ*2500 was observed to increase about threefold, as shown in Figure 9(c). Additionally, purified surfactin peaks for MS analysis from the mutant strain were observed to form a larger halo as compared to the wild-type strain (Figure 10); hence, the antimicrobial potential of the mutant was also enhanced compared to the parent strain. Likewise, purification through a TLC plate demonstrated the separation of multiple concentrated bands in mutant extract in contrast to the wild-type strain. The observation of surfactin peaks in our results within a mass-to-charge ratio range of 1000 linked to various isoforms differing with 14 Da of carbon in the hydroxyl fatty acid chain was in accordance with the results reported earlier [2].

## 5. Conclusions

This study reported comprehensive information on the successful development of *Bacillus* mutants through random mutagenesis using a combination of UV and gamma radiation with enhanced surfactin yield. The study is also novel as the combined use of both of the physical mutagen radiation sources to enhance surfactin production from *Bacillus* spp. has not been reported before. Moreover, the developed mutant AF-UV*γ*2500 also exhibited the increased ability to utilize the agroindustrial wastes as substrate without the pretreatment of it in addition to the enhanced relative abundance of surfactin and its isoforms as compared to the wild-type strain displayed in MALDI-TOF analysis with a potential impact as an antifungal compound. We are persuaded to conclude that this *Bacillus* strain is potent enough to utilize a variety of waste for the production of LPs, as the mutant produces 1.55 g/L surfactin at Erlenmeyer scale with corn steep liquor as carbon; if employed in a larger fermentation bioreactor, it can produce significant results. Due to the outcomes of using various agroindustrial wastes under optimized fermentation conditions, it proved to be a good potential alternative feedstock. The valorization of waste carbon sources can be exploited for future time course studies between the wild type and its mutant strain. Moreover, the refined nitrogen sources can be swapped with candy wastewater or nitrogenous cosmetic sludge to further cut the cost of fermentation medium at an industrial scale. Additionally, this study opens the pathway in an open research field: the application of the following bacterial strains to solid-state fermentation with waste residues as substrates for metabolite production.

## Figures and Tables

**Table 1:** Screening for wild-type and mutant strains for lipopeptide production.

***Bacillus* strains**	**Hemolytic assay (cm)**	**Oil spreading assay (cm)**	**Drop collapse assay**	**E**_24_	**Antifungal assay**
AF_3B	1.6 ± 0.042	2.5 ± 0.037	+	45	+++
OS2	1.2 ± 0.027	2.0 ± 0.024	+	37	++
BS9	1.1 ± 0.022	1.3 ± 0.018	+	33	+
OS2-UV*γ*2500	1.4 ± 0.057	2.4 ± 0.029	++	66	++
OS2-UV*γ*3000	1.5 ± 0.043	2.2 ± 0.032	+++	70	+++
AF-UV*γ*2500	1.8 ± 0.033	3.1 ± 0.057	+++	76	+++
AF-UV*γ*1500	1.67 ± 0.037	2.8 ± 0.030	+++	71	+++
Tween 80	—	—	—	70	—
Triton X-100	—	—	+++	—	—

*Note:* Antifungal activity: “+++”, excellent; “++”, good; “+”, fair, “—”, absent. Drop collapse assay: “+++”, drop collapse within 1 min; “++”, drop collapse after 1 min; “+”, drop collapse after 3 min of biosurfactant interaction. All experiments were run in triplicates.

**Table 2:** Optimization of carbon source for wild-type strain AF_3B and mutant strain AF-UV*γ*2500.

**Carbon source**	**Dry cell weight (g/L)**		**Surfactin yield (g/L)**		**Emulsification index**	
	**Wild-type AF**	**Mutant-AF-UV*γ*2500**	**Wild-type AF**	**Mutant AF-UV*γ*2500**	**Wild-type AF**	**Mutant AF-UV*γ*2500**
Sucrose	3.81 ± 0.0071	4.47 ± 0.071	0.65 ± 0.041	0.99 ± 0.043	46.12 ± 0.085	74.81 ± 0.68
Glucose	3.79 ± 0.021	3.81 ± 0.071	0.60 ± 0.023	0.77 ± 0.039	26.38 ± 0.035	58.34 ± 0.33
Maltose	3.63 ± 0.035	3.79 ± 0.085	0.41 ± 0.021	0.75 ± 0.040	26.11 ± 0.028	59.80 ± 0.41
Fructose	2.55 ± 0.014	3.61 ± 0.071	0.33 ± 0.016	0.58 ± 0.012	26.39 ± 0.099	49.85 ± 0.23
Lactose	3.60 ± 0.071	4.39 ± 0.042	0.58 ± 0.280	0.96 ± 0.07	25.82 ± 0.028	64.11 ± 0.69
Glycerol	2.49 ± 0.014	4.98 ± 0.028	0.30 ± 0.016	1.02 ± 0.012	26.32 ± 0.057	76.43 ± 0.74
Starch	2.52 ± 0.028	3.09 ± 0.028	0.43 ± 0.031	0.72 ± 0.016	40.49 ± 0.057	61.00 ± 0.43

*Note:* Values presented are as the mean ± standard deviation (*n* = 3).

**Table 3:** Optimization of nitrogen source for wild-type strain AF_3B and mutant strain AF-UV*γ*2500.

**Nitrogen source**	**Dry cell weight (g/L)**		**Surfactin yield (g/L)**		**Emulsification index (** **E**_24_ **)**	
	**Wild-type AF**	**Mutant AF-UV*γ*2500**	**Wild-type AF**	**Mutant AF-UV*γ*2500**	**Wild-type AF**	**Mutant AF-UV*γ*2500**
Beef extract	2.80 ± 0.027	4.18 ± 0.071	0.62 ± 0.030	0.94 ± 0.053	36.12 ± 1.35	55.81 ± 1.68
Yeast extract	3.88 ± 0.030	4.97 ± 0.085	0.75 ± 0.021	0.43 ± 0.056	51.32 ± 1.55	65.76 ± 1.95
Tryptone	2.56 ± 0.021	5.25 ± 0.030	0.67 ± 0.023	1.55 ± 0.061	26.38 ± 1.27	82.28 ± 2.11
Peptone	2.43 ± 0.035	3.72 ± 0.025	0.61 ± 0.021	0.75 ± 0.030	32.11 ± 1.28	59.80 ± 1.41
Urea	1.55 ± 0.013	3.61 ± 0.071	0.33 ± 0.016	0.58 ± 0.029	24.39 ± 1.09	49.85 ± 1.23
(NH_4_)_2_SO_4_	3.71 ± 0.011	4.22 ± 0.042	0.69 ± 0.031	1.08 ± 0.031	42.32 ± 1.88	72.11 ± 1.69
(NH_4_)_2_NO_3_	1.59 ± 0.014	4.28 ± 0.029	0.30 ± 0.016	0.98 ± 0.035	26.32 ± 1.05	76.43 ± 2.09
NH_4_CL	1.93 ± 0.028	3.59 ± 0.025	0.41 ± 0.021	0.52 ± 0.029	22.49 ± 1.00	41.00 ± 1.23
NaNO_3_	2.52 ± 0.023	3.69 ± 0.026	0.50 ± 0.026	0.67 ± 0.033	27.49 ± 1.05	51.00 ± 1.33

*Note:* Values presented are as the mean ± standard deviation (*n* = 3).

## Data Availability

The sequencing data for the 16S rRNA gene of the three strains used in this study has been deposited to NCBI under Accession Numbers OL771696.1, OL771697.1, and OL757572.1 for AF_3B, OS2, and BS9, respectively.
