# Supplementary material for: Application of Combined Irradiation Mutagenesis Technique for Hyperproduction of Surfactin in Bacillus velezensis Strain AF_3B
Source: Int J Microbiol. 2025 Feb 20;2025:5570585. doi: 10.1155/ijm/5570585 (PMC11867730; doi:10.1155/ijm/5570585)
Supplement: Supporting Information — Additional supporting information can be found online in the Supporting Information section. Table S1: Screening for wild-type and mutant strains for lipopeptide production. [file 5570585.f1.docx]

**Table S1** Screening for wild type and mutant strains for lipopeptide production.

| **Isolate code** | **Mutant code** | **colony morphology** | **Oil spreading assay** | **Hemolytic assay** | **Drop collapse assay** |
| --- | --- | --- | --- | --- | --- |
| AF-UV | AF-UVγ100 | Opaque white, circular and smooth margins | 2.3±0.029 | 1.58±0.033 | ++ |
|  | AF-UVγ100-IV |  | 2.3±0.036 | 1.59±0.037 | +++ |
|  | AF-UVγ300-V |  | 2.5±0.029 | 1.6±0.031 | +++ |
|  | AF-UVγ500 |  | 2.5±0.031 | 1.58±0.029 | +++ |
|  | AF-UVγ500-II |  | 2.4±0.029 | 1.61±0.033 | +++ |
|  | AF-UVγ500-VI |  | 2.5±0.028 | 1.6±0.032 | +++ |
|  | AF-UV1000 |  | 2.6±0.031 | 1.62±0.033 | +++ |
|  | AF-UVγ1000-V |  | 2.5±0.033 | 1.62±0.033 | +++ |
|  | AF-UVγ1000-VI |  | 2.6±0.031 | 1.62±0.033 | +++ |
|  | AF-UVγ1000-VII |  | 2.6±0.030 | 1.61±0.031 | +++ |
|  | AF-UVγ1200-II |  | 2.6±0.032 | 1.62±0.035 | +++ |
|  | AF-UVγ1200-VI |  | 2.7±0.031 | 1.63±0.033 | +++ |
|  | AF-UVγ1500 | Opaque, raised margins | 2.8±0.030 | 1.67±0.037 | +++ |
|  | AF-UVγ1500-VII |  | 2.8±0.030 | 1.67±0.033 | +++ |
|  | AF-UVγ1500-IX |  | 2.8±0.032 | 1.6±0.031 | +++ |
|  | AF-UVγ2000 |  | 2.7±0.031 | 1.54±0.032 | +++ |
|  | AF-UVγ2000-II |  | 2.5±0.032 | 1.53±0.030 | +++ |
|  | AF-UVγ2000-V |  | 2.7±0.032 | 1.5±0.036 | +++ |
|  | AF-UVγ2000-VI |  | 2.6±0.037 | 1.52±0.031 | +++ |
|  | AF-UVγ2500 |  | 3.1±0.057 | 1.8±0.033 | +++ |
|  | AF-UVγ2500-I |  | 3.1±0.041 | 1.8±0.031 | +++ |
|  | AF-UVγ2500-III |  | 3.1±0.047 | 1.8±0.037 | +++ |
|  | AF-UVγ2500-VII |  | 3.2±0.036 | 1.8±0.033 | +++ |
|  | AF-UVγ2500-IX |  | 3.1±0.031 | 1.78±0.033 | +++ |
|  | AF-UVγ2500-X |  | 3.2±0.033 | 1.8±0.030 | +++ |
|  | AF-UVγ3000 |  | 3.0±0.031 | 1.78±0.031 | +++ |
|  | AF-UVγ3000-I |  | 3.1±0.047 | 1.69±0.033 | +++ |
|  | AF-UVγ3000-IV |  | 3.2±0.043 | 1.68±0.032 | +++ |
|  | AF-UVγ3000-V |  | 3.2±0.041 | 1.6±0.031 | +++ |
|  | AF-UVγ3000-IX |  | 3.1±0.041 | 1.7±0.031 | +++ |
| OS2-UV | OS2-UVγ100 | Opaque, smooth and circular colony | 2.1±0.021 | 1.2±0.029 | ++ |
|  | OS2-UVγ300 |  | 2.1±0.027 | 1.2±0.030 | ++ |
|  | OS2-UVγ300-II |  | 2.1±0.024 | 1.2±0.031 | ++ |
|  | OS2-UVγ500 |  | 2.1±0.022 | 1.2±0.033 | ++ |
|  | OS2-UVγ1000 |  | 2.1±0.021 | 1.25±0.037 | ++ |
|  | OS2-UVγ1000-V |  | 2.2±0.023 | 1.29±0.031 | ++ |
|  | OS2-UVγ1200 |  | 2.2±0.021 | 1.25±0.033 | ++ |
|  | OS2-UVγ1500-III |  | 2.3±0.026 | 1.29±0.035 | ++ |
|  | OS2-UVγ2000 |  | 2.3±0.027 | 1.31±0.032 | +++ |
|  | OS2-UVγ2000-IX |  | 2.3±0.022 | 1.3±0.038 | +++ |
|  | OS2-UVγ2000-X |  | 2.3±0.022 | 1.3±0.031 | +++ |
|  | OS2-UVγ2500 | Opaque embedded colony with raised margins | 2.4±0.029 | 1.4±0.057 | ++ |
|  | OS2-UVγ2500-II |  | 2.3±0.032 | 1.5±0.035 | ++ |
|  | OS2-UVγ3000 |  | 2.2±0.032 | 1.5±0.043 | +++ |
|  | OS2-UVγ3000-VI |  | 2.2±0.031 | 1.5±0.035 | +++ |
| BS9 | BS9-γ1200 | Translucent to clear colony with sticky and smooth margins | 1.5±0.025 | 1.0±0.021 | ++ |
|  | BS9-γ1500 |  | 1.5±0.025 | 1.3±0.021 | ++ |
|  | BS9-γ1500-I |  | 1.5±0.021 | 1.3±0.031 | + |
|  | BS9-γ2500 |  | 1.6±0.032 | 1.5±0.023 | ++ |
|  | BS9-γ3000 |  | 1.6±0.029 | 1.6±0.021 | ++ |

Drop collapse assay: ‘+++’- drop collapse within 1 minute, ‘++’- drop collapse after 1 minute, ‘+’- drop collapse after 3 minutes of biosurfactant interaction. All experiments were run in triplicates.
